# Correlating Capsaicinoid Levels and Physicochemical Properties of Kimchi and Its Perceived Spiciness

**DOI:** 10.3390/foods10010086

**Published:** 2021-01-04

**Authors:** So-Ra Yoon, Yun-Mi Dang, Su-Yeon Kim, Su-Yeon You, Mina K. Kim, Ji-Hyoung Ha

**Affiliations:** 1 Hygienic Safety and Analysis Center, World Institute of Kimchi, Gwangju 61755, Korea; sorayoon@wikim.re.kr (S.-R.Y.); ymdang@wikim.re.kr (Y.-M.D.); kss1112@wikim.re.kr (S.-Y.K.); ysy@wikim.re.kr (S.-Y.Y.); 2 Department of Food Science and Human Nutrition, Jeonbuk National University, Jeollabuk-do 54896, Korea

**Keywords:** capsaicinoid, kimchi, partial correlation, Pearson’s correlation, spiciness

## Abstract

Capsaicinoid content, among other factors, affects the perception of spiciness of commercial kimchi. Here, we investigated whether the physicochemical properties of kimchi affect the spicy taste of capsaicinoids perceived by the tasting. High-performance liquid chromatography (HPLC) was used to evaluate the capsaicinoid content (mg/kg) of thirteen types of commercial kimchi. The physicochemical properties such as pH, titratable acidity, salinity, free sugar content, and free amino acid content were evaluated, and the spicy strength grade was determined by selected panel to analyze the correlation between these properties. Panels were trained for 48 h prior to actual evaluation by panel leaders trained for over 1000 h according to the Spectrum^TM^ method. Partial correlation analysis was performed to examine other candidate parameters that interfere with the sensory evaluation of spiciness and capsaicinoid content. To express the specific variance after eliminating the effects of other variables, partial correlations were used to estimate the relationships between two variables. We observed a strong correlation between spiciness intensity ratings and capsaicinoid content, with a Pearson’s correlation coefficient of 0.78 at *p* ≤ 0.001. However, other specific variables may have influenced the relationship between spiciness intensity and total capsaicinoid content. Partial correlation analysis indicated that the free sugar content most strongly affected the relationship between spiciness intensity and capsaicinoid content, showing the largest first-order partial correlation coefficient (r_xy/z_: 0.091, *p* ≤ 0.01).

## 1. Introduction

Kimchi is traditional Korean food made by fermenting leafy vegetables. Kimchi has been listed in the Codex Alimentarius, 2001 (CODEX STAN 223-2001) and was listed in 2006 as one of the five healthiest foods worldwide [1]. Among the various fermented vegetables available in Asian countries such as *Zacái*, *Yancái*, and *Suncái* in China and *Tsukemono* in Japan [2], Korean-style kimchi contains red pepper (*Capsicum annuum*) powder and various seasonings, which impart its distinctive pungent-spicy characteristics [3]. Because of its distinctive pungent-spicy perception, red pepper powder is one of the most accepted spices worldwide [4]. Although spiciness is not scientifically considered as a gustative stimulus, unlike sweet, salty, sour, bitter, and umami tastes from cultural perspectives, Asian countries consider the pungent-spicy stimulus produced by red pepper powder as one of the six main taste modalities [5]. The pungent-spicy characteristics of Korean-style kimchi drive consumer preferences among Chinese [6] and American consumers [7,8]. Park et al. [8] reported an increase in the perception of spiciness in kimchi containing increased levels of red pepper powder in the formulation, indicating that red pepper powder is a key contributor to the spicy taste in kimchi.

As the spicy taste attributed to red pepper powder in kimchi has been identified as one of the key factors influencing consumer preference, a study to determine the spiciness of kimchi was previously conducted [8]. To date, determining spiciness in a food matrix, including kimchi and red pepper powder, depends on the instrumental analysis of capsaicinoids. The levels of capsaicin (CAP) and dihydrocapsaicin (DHCA), which are major components of red pepper powder, can be assessed quantitatively and qualitatively through various assays and analytical instruments. Initially, pepper spiciness was measured using the Scoville organoleptic heat test [9]. In addition to determining the Scoville unit (SU), spectrophotometry [10], colorimetry [11], high-performance liquid chromatography (HPLC) [12], gas chromatography [13], liquid chromatography [14], liquid chromatography mass spectrometry [15], and capillary electrophoresis [16] have been used to analyze the contents of CAP and DHCA. HPLC is a widely used method for precisely quantifying CAP and DHCA [12]. Based on literature reviews, instrumental analysis of CAP and DHCA in food matrices revealed possible interactions between CAP and other quality characteristics such as soluble sugars, organic acids, and amino acids in red peppers [17] and between organic compounds and capsaicinoid content in red pepper [18]. When considering kimchi fermentation, accumulation of organic acids such as lactic acids [7] decreases the pH of kimchi to 4.2, potentially influencing its pungent-spicy taste.

Although the abovementioned methods provide the concentrations of CAP and DHCA in food matrices, information on the perceived spiciness from human sensory perspectives is lacking. To date, few studies have examined the pungent-spicy taste in kimchi using human assessors. Chambers et al. [19] reported a sensory lexicon describing commercially available cabbage (*Baechu*) kimchi. They used 0.4 mg/kg capsaicin solution as a reference for “heartburn” and Reese’s horseradish sauce as a reference for the “pungent” sensation in kimchi. Similarly, Ku et al. [9] investigated the physicochemical and sensory characteristics of radish-based kimchi (*Kakdugi*) made by red pepper and red pimiento and reported a high correlation between CAP and DHCA and a “hot taste” in kimchi. Instead of solely investigating the spiciness characteristics in kimchi as perceived by human assessors, they used “heat burn”, “pungent”, and/or “hot taste” as part of the sensory lexicon describing the sensory characteristics of kimchi. Therefore, perceived spiciness from a human sensory perspective remains unclear. Park et al. [8] reported the consumer acceptance of kimchi containing varying levels of red pepper and fish sauce. They found that the ratings of perceived spiciness increased with the levels of red pepper powder and overall consumer acceptability increased with the spiciness of kimchi. However, they used the percentage of red pepper in kimchi as an indicator of spiciness; therefore, the perceived spiciness by a human was not objectively profiled.

Although many factors influence the human perception of spiciness in kimchi, a sensory analysis protocol has not been developed for evaluating the spiciness of kimchi using a trained panel. In this study, we examined whether sensory spiciness caused by capsaicinoids is influenced by the specific physicochemical properties of kimchi such as pH, titratable acidity, salinity, free sugars, and free amino acids. Additionally, we used a partial correlation approach to examine other candidate variables that interfere with two closely related variables; this approach was used to determine the causes of variation in human sensory perception of the intensity of pungent stimuli. Partial correlations were used to estimate the correlation between two variables and define the specific variance explained by eliminating the effect of other variables.

## 2. Materials and Methods

### 2.1. Kimchi Samples

Thirteen types of kimchi, produced by various manufacturers and with known variations in spiciness and CAP concentrations, were purchased from grocery stores in Gwangju, Korea. All ingredients in the kimchi samples are listed in Table 1 based on the package labeling. Upon purchase, all kimchi samples were blended (Philips HR1372, Amsterdam, The Netherlands) and immediately analyzed.

### 2.2. Determination of Physicochemical Properties of Kimchi

#### 2.2.1. Assessment of Capsaicin and Dihydrocapsaicin Content

The CAP and DHCA contents were analyzed as previously described [20] with minor modifications. Briefly, 15 mL methanol was added to a vial containing 2.5 g of homogenized kimchi and two or three glass beads. The mixture was placed on a heating block at 90 °C for 1 h and then cooled. The cooled extract was passed through a filter paper (8 μm, Whatman No. 2, Kent, UK), and methanol was added to the filtered solution to bring the volume to 25 mL; the solution was filtered again through a 0.2-μm syringe filter (Millipore, Billerica, MA, USA). The analysis was conducted using HPLC Model 1260 Infinity; Agilent Technologies, Santa Clara, CA, USA) coupled with a fluorescence detector (Agilent Technologies, Santa Clara, CA, USA). Excitation and emission wavelengths were set to 208 and 325 nm, respectively. To separate CAP and DHCA, a Lachrom Ultra C18 column (2 × 50 mm, 2 μL; Hitachi, Tokyo, Japan) was used with 0.1% acetic acid and acetonitrile (6:4, *v*/*v*) as mobile phase at a flow rate of 0.6 mL/min with an injection volume of 2 μL.

#### 2.2.2. pH and Titratable Acidity

The homogenized kimchi samples were filtered to obtain the filtrate used to measure the pH and titratable acidity. pH was measured using a pH meter (Model Titro Line 5000; SI Analytics, Mainz, Germany). The kimchi was titrated to pH 8.3 by adding 0.1 N sodium hydroxide (NaOH; Daejung Chemical, Shiheung, Korea) solution, and titratable acidity was calculated as the percentage of lactic acid using Equation (1).

(1) $$Titratable\;acidity\;\left(\%\right)=\;\frac{\left(A\;\times F\times D\right)\times 0.009}{S}\;\times 100$$

where *A* is the titration volume (mL) of 0.1 N NaOH; *F* is the titer of 0.1 N NaOH; *D* is the dilution factor of the sample; 0.009 is lactic acid corresponding to 1 mL 0.1 N NaOH; and *S* is sample weight (g).

#### 2.2.3. Salinity

Salinity was measured using the Mohr method [21]. Sodium chloride, which reacts with silver nitrate, is precipitated to silver chloride and acquires a reddish-brown color by reacting with potassium chromate. To measure kimchi salinity, 2.5 g homogenized kimchi were combined with 47.5 mL distilled water and passed through filter paper (8 mm, Whatman No. 2). Next, 200 μL of 10% potassium chromate were added to 10 mL filtrate and titrated with 0.02 N silver nitrate until the color of the mixture changed from clear yellow to reddish-brown. Salinity was calculated using Equation (2).

(2) $$Salinity\;\left(\%\right)=\;\frac{\left(A\;\times F\times D\right)\times 0.00117}{S}\;\times 100$$

where *A* is the titration volume of 0.02 N silver nitrate (mL); *F* is the titer of 0.02 N silver nitrate; *D* is the dilution factor; 0.00117 is the sodium chloride corresponding to 1 mL 0.02 N silver nitrate; and *S* is the sample weight (g).

#### 2.2.4. Determination of Free Sugar Content

After filtering the homogenized kimchi samples through sterilized gauze, the obtained kimchi juice (filtrate) was extracted in a water bath at 85 °C for 25 min, cooled to room temperature (18 ± 2 °C), and passed through a 0.2-μm syringe filter. Analysis was performed using HPLC equipped with a refractive index detector. To separate the sugars (glucose, fructose, sucrose, maltose, mannitol, and sorbitol), we used an Asahipak NH2P-504 E column (250 × 4.6 mm; Shodex, Tokyo, Japan) with acetonitrile (75%, *v*/*v*) as the mobile phase at a flow rate of 1 mL/min using a described protocol [22].

#### 2.2.5. Determination of Free Amino Acid Content

To extract free amino acids, each homogenized kimchi sample (1 g) was mixed with 5 mL of 5% trichloroacetic acid and centrifuged at 8000× *g* for 20 min. Next, 1 mL supernatant was combined with 0.02 N hydrochloric acid and passed through a 0.2-μm syringe filter. An automatic amino acid analyzer (Model L-8900; Hitachi, Tokyo, Japan), equipped with an ion-exchange column (4.6 × 60 nm HPLC pack column and #2622 SCF PF column; Hitachi, Tokyo, Japan), and L-8900 buffer solution (Wako Pure Chemical Industries, Osaka, Japan) were used to analyze the free amino acid content. The flow rate was 0.35 mL/min, and absorbance was set to 440 and 570 nm.

### 2.3. Descriptive Analysis of the Spiciness of Kimchi

#### 2.3.1. Panel Selection

Descriptive analysis was conducted following the ethical guidelines of the Helsinki Declaration and signed informed consent was obtained from all participants of the descriptive sensory panel before evaluation. Participants in the panel were recruited from the World Institute of Kimchi (Gwangju, Korea) based on their interests and experience in sensory evaluation. The enrolled participants were assessed for their ability to discriminate the five tastes (sweet, salt, sour, bitter, and umami), aroma recognition, and spiciness tolerance. Those who passed the screening test participated in the panel training. The selected panel comprised six participants, including a panel leader, aged from 27 to 39 years (1 male and 5 females).

#### 2.3.2. Reference Sample Evaluation

To establish a frame of reference for the range of sample concentrations, capsaicin solutions of various concentrations were prepared according to the method of Lee and Kim [20]. Briefly, 0.02 g of 95% capsaicin (Sigma-Aldrich, St Louis, MO, USA) was mixed with 0.7 g food-grade polysorbate-80 (Sigma-Aldrich, St Louis, MO, USA) and dissolved by heating on a hot plate (40 ± 2 °C). After cooling, the mixture was adjusted to a volume of 100 mL by adding water at a temperature of 70 °C. The prepared capsaicin stock solution (200 mg/kg) was diluted to various concentrations (from 0.5 to 30.0 ppm, *w*/*v*), and served as the sensory reference for pungent-spicy taste in kimchi.

#### 2.3.3. Panel Training

The panel was trained for 48 h before the actual evaluation. The panel leader had more than 1000 h of training in the evaluation of various food products using the Universal scale in the Spectrum^TM^ method. Panel training was performed following the ASTM method of descriptive analysis testing for sensory evaluation (ASTM: MNL13) [23]. To maximize the evaluation ability, panelists were instructed to avoid food with strong aroma and flavor, coffee, and alcoholic beverages for 2 h before the evaluation. All participants were familiar with commercial kimchi and with the spiciness taste in kimchi. Before evaluation, panelists were familiarized with the different types of commercial kimchi. During training, panelists were taught the Universal scale in the Spectrum^TM^ method using basic taste solutions such as sucrose for sweet, salt for salty, citric acid for sour, caffeine for bitter, and monosodium glutamate for umami tastes [24]. Next, they were trained to understand the rating system for spiciness using capsaicin solution (2 = 0.5 mg/kg capsaicin solution; 4 = 2.0 mg/kg capsaicin solution; 7 = 4.0 mg/kg capsaicin solution; 9 = 6.0 mg/kg capsaicin solution). During training, the sample evaluation protocol for the spiciness taste of kimchi and palate cleanser for removing residual spiciness in the mouth were evaluated. To evaluate spiciness using capsaicin solution, 10 mL of capsaicin solution was consumed and rinsed around the palate for 10 s before expectoration; the intensity of spiciness was rated within 10 s. Participants were then asked to use whipping cream with 5% sucrose (22 ± 2 °C, *w*/*v*) unsalted crackers (5 g), and water (22 ± 2 °C, *w*/*v*) as a primary palate cleanser. In addition to the cleansing protocol, a 10-min resting gap period between tasting of each sample was enforced.

#### 2.3.4. Kimchi Sample Evaluation

All samples (10 g each) were served in a white plastic cup (diameter: 70 mm, length: 35 mm) marked with a three-digit number generated in random order for each evaluation. Kimchi was served to the panel in a blended form to minimize part-by-part variations (i.e., leafy part and stem part of kimchi) in spiciness. Evaluations were conducted in a sensory booth at room temperature (18 ± 2 °C). Before evaluating the spiciness of kimchi, the panelists calibrated their palate using capsaicin reference [0.5, 2.0, 4.0, 6.0 ppm (*w*/*v*)] in ascending order. The trained panelists rated the spiciness of each kimchi sample individually, and the orders of presentation of kimchi samples among panelists followed a randomized and balanced design.

### 2.4. Analysis Using Simple and Partial Correlation Coefficients

If the correlation is explicitly defined between *X* and *Y* without conditioning on any variable, the order of the partial correlation coefficient is zero. This can generally be defined as Pearson’s correlation (Equation (3)). The order is x when the correlation is calculated after conditioning on x number of different variables other than A and B [25]. Equation (4) generally defines the first three orders of partial correlations.

Zero-order correlation:

(3) $$r_{XY}=\;\frac{cov\left(X,Y\right)}{\sqrt{var\left(X\right)\times var\left(Y\right)}}$$

First-order partial correlation:

(4) $$r_{XY/Z}=\;\frac{\left[r_{XY}-\left(r_{YZ}\;\times \;r_{XZ}\right)\right]}{\sqrt{\left(1-r_{YZ}^{2}\right)\left(1-r_{XZ}^{2}\right)}}$$

where *r_xy/z_* is the correlation coefficient suspected of being affected by specific variables (*Z*: each physicochemical property such as pH, titratable acidity, salinity, free sugars, and free amino acids); *r_yz_* is the correlation of corresponding specific variables with the rating of spiciness; and *r_xz_* is the correlation of the capsaicinoid content variable with specific variables (*Z*).

### 2.5. Statistical Analysis

All results are presented as the mean ± standard deviation. Data were analyzed using SPSS v.19 for Windows (SPSS, Inc., Chicago, IL, USA). Analysis of variance was used to verify the significance of differences among the physicochemical properties of kimchi samples regarding the spiciness intensity of each sample. Correlations were analyzed using R program (http://www.r-project.org). *p* < 0.05 was considered as statistically significant.

## 3. Results and Discussion

### 3.1. Physicochemical Properties of Commercial Kimchi

The ingredients and fermentation period of each kimchi sample varied; therefore, the sensory attributes of these samples differed, necessitating assessment of the correlation between spiciness and physicochemical components. Detection and quantitative assessment of the capsaicin content were used to profile the spiciness-related characteristics of commercial kimchi. Except for salinity, most physicochemical properties of kimchi such as pH, titratable acidity, free sugars, and free amino acids change under conditions of lactic acid fermentation [26]. Therefore, as the commercial kimchi products were purchased immediately after manufacture, maturity among kimchi samples did not influence the results.

Physicochemical properties such as pH, titratable acidity, salinity, free sugar content, and free amino acid content of kimchi samples are shown in Table 2. The pH values of all kimchi samples widely varied from 4.13 to 6.32. Commercial kimchi generally has various pH values between 4.0 and 6.0 when freshly prepared following the manufacturers’ individual recipes; this value gradually decreases because of organic acids generated by lactic acid bacteria during fermentation [27,28]. The titratable acidities (TAs) of all kimchi samples ranged from 0.27 to 0.84 for unfermented samples of packaged kimchi. Previous studies reported that commercial kimchi has optimum flavor and texture attributes at a pH of 4.5 and/or TA of 0.6–0.9% [29,30]. Several samples in this study met the criteria for optimum flavor and texture attributes (Samples 4, 5, 10, and 12); however, our study did not focus on the flavor and texture of kimchi but rather on its spiciness. Overall, the pH and TAs of our kimchi samples were within the range of those of reported commercial kimchi samples [27,29]. The salinity of kimchi samples was between 1.34% and 2.09%. According to Jung et al. [31], free sugar content plays a major role in the flavor development of kimchi, as it alters the composition of lactic acid bacterial communities, which generates a sweet taste. Table 2 shows that the free sugar content in our kimchi samples ranged from 19.92 to 52.24 mg/mL. Among free sugars, fructose and glucose accounted for most of the sugar content in our kimchi samples at 44.07% and 41.37%, respectively, regarding the total free sugar content (Table S1). The content of mannitol, sucrose, maltose, and sorbitol in our commercial kimchi samples was below 15%. Free amino acids in kimchi, which are also known to contribute to kimchi flavor, were analyzed. Among the 40 free amino acids, 32 varied in their concentration among the commercial kimchi products examined in this study. Glutamine, glutamic acid, alanine, and γ-aminobutyric acid were generally predominant in our kimchi samples, accounting for approximately 47% of the total free amino acid content (Table S2). The generation of glutamic acid in kimchi is caused either by glutaminase activity or by proteolysis of glutamine by lactic acid bacteria [32].

### 3.2. Capsaicinoid Content of 13 Commercial Kimchi Samples

**Figure 1:**
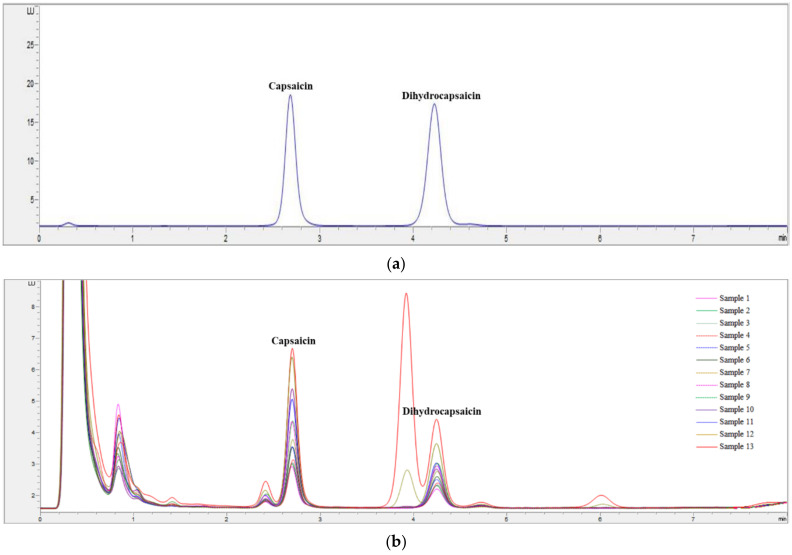
High-performance liquid chromatography (HPLC) chromatograms of: (**a**) a standard solution containing capsaicin and dihydrocapsaicin; and (**b**) capsaicin and dihydrocapsaicin in 13 commercial kimchi samples.

**Figure 2:**
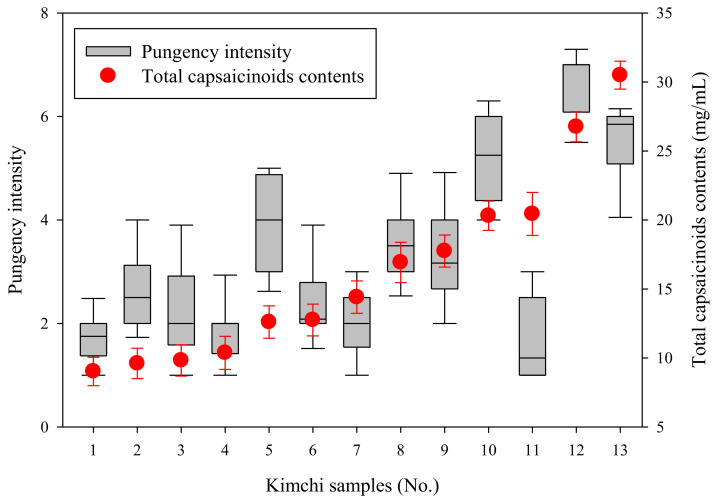
Sensory spiciness evaluation of commercial kimchi samples (left *Y*-axis). Total capsaicinoid content of commercial kimchi samples (right *Y*-axis). The results of sensory evaluation assessing the spiciness and mean capsaicinoid content of our kimchi samples are represented in box plots and scatter plots, respectively. The box plots show the lowest, median, and highest data points, as well as the spread and distribution of spiciness intensity values.

Capsaicinoid, which is one compound responsible for pungent taste, provides an irritating stimulus and is the principal chemical component of red pepper powder used to impart the pungent taste to kimchi. CAP and DHCA account for most capsaicinoid content, and a previous study reported that CAP and DHCA contribute equally to the spiciness intensity [33]. Therefore, total capsaicinoid, which included CAP and DHCA, was reported as an instrumental analysis of the spiciness of commercial kimchi samples (Table 3). A representative chromatogram of CAP and DHCA reference standards, along with their retention times, is shown in Figure 1. The retention times of CAP and DHCA were 2.7 and 4.7 min, respectively. The total capsaicinoid content of the 13 kimchi samples used in our study ranged from 9.02 to 30.50 mg/kg (Table 3). As shown in Table 3, our results indicate that spiciness did not depend exclusively on the mean total capsaicinoid content; additional factors, other than total capsaicinoid content, influenced the spiciness intensity of kimchi (Figure 2).

### 3.3. Sensory Evaluation of Spiciness of Commercial Kimchi

The spiciness intensities of our kimchi samples corresponding to that of capsaicin solutions of 0.5, 2.0, 4.0, and 6.0 ppm (*w*/*v*) were denoted as scores of “1”, “4”, “7”, and “9”, respectively. Among our kimchi samples, the lowest spiciness intensity (1) was similar to that of 0.5 ppm (*w*/*v*) capsaicin solution, whereas the maximum (9) spiciness intensity was similar to that of 6.0 ppm (*w*/*v*) capsaicin concentration. This maximum level was slightly higher than the spiciest kimchi samples used in our study. None of our kimchi samples exceeded the spiciness intensity of 6.0 ppm (*w*/*v*) capsaicin solution. The mean values of spiciness intensity were estimated using the results obtained from individual participants. The change in the values for spiciness intensity and mean total capsaicinoid content indicated a direct relationship between spiciness intensity and mean total capsaicinoid content. According to Schneider et al. [34], the distribution of values in a box plot can be explained by individual differences in the spiciness perception of the panelists, which is then reflected in the assigned spiciness intensity scores. Even kimchi samples with mostly the same mean total capsaicinoid content often differed in sensory spiciness ratings. For example, the total capsaicinoid content of Sample 11 was nearly twice that of Sample 4; however, the sensory spiciness ratings of Samples 4 and 11 did not differ significantly. Furthermore, the total capsaicinoid contents of Samples 10 and 11 were similar, whereas their sensory spiciness ratings differed significantly. These results indicate that factors such as the physicochemical properties of kimchi can influence the perception of spiciness.

### 3.4. Correlation between Physicochemical Parameters and Spiciness

**Figure 3:**
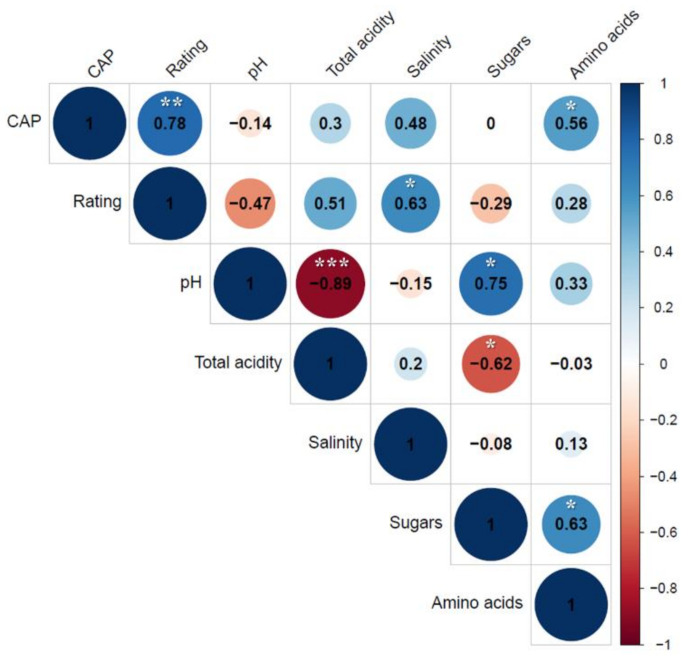
Correlation coefficient matrix of sensory spiciness evaluation and physicochemical parameters in commercial kimchi samples. Positive coefficients are represented by blue circles, which indicate a direct relationship between variables in the matrix, and negative coefficients are shown as red circles, which reflect an inverse relationship. * *p* ≤ 0.05, ** *p* ≤ 0.01, *** *p* ≤ 0.001.

To confirm the results shown in Table 3, Pearson’s correlation coefficient (r) (Figure 3) was analyzed. Among the seven factors, four (spiciness intensity rating, total acidity, salinity, and free amino acids) were positively correlated with an increase in capsaicinoid content; one factor (pH) was negatively correlated with the capsaicinoid content; and zero correlations were observed between the free sugar content and capsaicinoid content.

We observed a strong relationship of 0.78 at *p* ≤ 0.01 between spiciness intensity ratings and the capsaicinoid content. Although we investigated the overall correlation coefficients between physicochemical properties and spiciness intensity ratings, our results suggest that physicochemical properties affect the taste of spiciness of kimchi. Pearson’s correlation coefficient (r) is widely used to determine the linear relationships between continuous random factors [35]. However, this value alone cannot differentiate the effects of direct and indirect associations between various factors. Correlation is a statistical technique used to calculate Pearson’s correlation between independent and dependent variables while controlling/eliminating the effects of one or more other correlation variables. Hence, partial correlations are used in many studies pertaining to medicine, metabolomics, genomics, and cognitive psychology.

**Figure 4:**
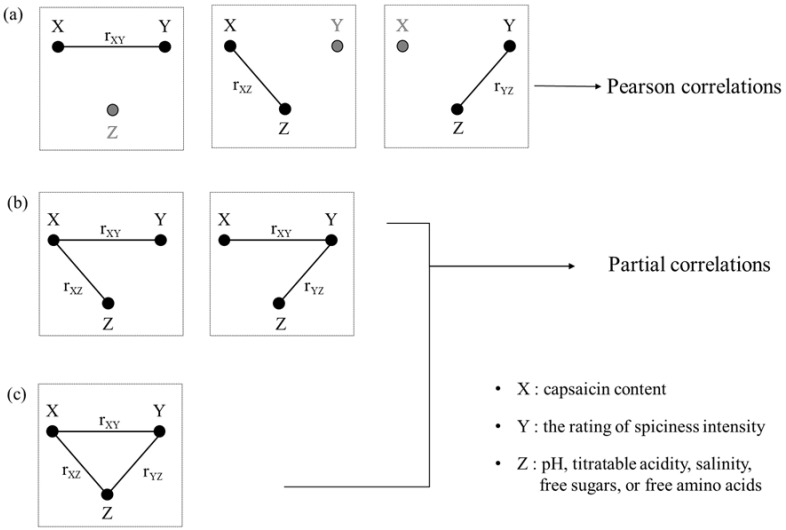
Graphical illustration of Pearson’s and partial correlations among the three random variables X, Y, and Z: (**a**) Pearson correlations; (**b**) and (**c**) partial correlations.

According to Melissa et al. [36], an association between variables X and Y can occur in various manners, such as a direct relationship X→Y co-regulated by a third variable Z (i.e., Z→X and Z→Y), or as an indirect relationship X→Z→Y. These relationships are shown in Figure 4, which illustrates how: (a) variable Z is not correlated with both variables X and Y; (b) only the random variable Y is correlated with variable Z; and (c) variable Z is correlated with both variables X and Y. Figure 4a shows that variable Z is independent of both variable X and variable Y; thus, both Pearson’s correlation coefficient and the partial correlation coefficient should theoretically be identical. Because variable Z correlates with variable X and variable Y in Figure 4b,c, a partial correlation is established. When variable X alone is correlated with Z, as illustrated in Figure 4b, Pearson’s correlation coefficients of variables X and Y differ depending on the influence of variable Z on variable X. Figure 4c shows that the two correlation coefficients are dissimilar.

In this study, we used a partial correlation approach to investigate parameters that interfere with two closely related parameters and determine the causes for variation in the human sensory perception of spiciness intensity. The partial correlation coefficient of variable X (capsaicin content) and variable Y (rating of spiciness intensity) controlled the influence of variable Z (pH, titratable acidity, salinity, free sugars, or free amino acids). The obtained first-order partial correlation coefficient (r_xy/z_) is shown in Table 4. Pearson’s correlation coefficient between spiciness intensity rating and capsaicinoid content was 0.777 at *p* ≤ 0.01, whereas the first-order partial correlation coefficients (r_xy/z_), calculated using Eq. 4, were 0.814 (pH), 0.762 (titratable acidity), 0.694 (salinity), 0.901 (free sugars), and 0.778 (free amino acids) (Table 4). Interestingly, the first-order partial correlation coefficient (r_xy/z_) showed the greatest differential of 0.901 at *p* ≤ 0.01. The first-order partial correlation coefficient differed from Pearson’s correlation coefficient because the calculated numerical difference between “r_xy/z_” [first-order partial correlation coefficient of capsaicin content (X) and rating of spiciness intensity (Y) without the influence of free sugar content (Z)] and “r_xy/z_” [Pearson’s correlation coefficient for capsaicin content (X) and rating of spiciness intensity (Y)] was influenced by the free sugar content as the conditioned variable Z. If the partial correlation coefficient differs significantly from the simple-correlation coefficient, the controlled variable (Z) is strongly related to the other two variables. Regarding the influence of sugar components as neutralizers of the spiciness of chili peppers, Stevens and Lawless [37] demonstrated that sugars are highly effective for decreasing spiciness intensity in the mouth. Schneider et al. [33] reported that the low rating for the sensory spiciness of commercial salsas may be due to sugars. Furthermore, it is well known that the effects of palate cleansers are markedly increased by addition of sucrose. These results were supported by various studies showing the effects of sucrose [38,39]. According to Steiner et al. [36], sweet taste is known to cause pleasant emotions in people of all ages, and sucrose allows people and animals to endure pain better when they taste spiciness [39,40]. This notion supports our finding that the free sugar content exerted the greatest effect on the relationship between spiciness intensity rating and capsaicin content of commercial kimchi.

## 4. Conclusions

Using partial correlation coefficients, we demonstrated that the sensory spiciness caused by capsaicinoids is influenced by the physicochemical properties of kimchi such as pH, titratable acidity, salinity, free sugars, and free amino acids. Among the investigated physicochemical properties of kimchi, free sugar content exerted the greatest direct and indirect effects on the rating of spiciness intensity. Our findings suggest that the capsaicinoid content cannot be used exclusively to rate the spiciness of commercial kimchi.

## Figures and Tables

**Table 1:** Ingredients in 13 commercial kimchi samples in this study.

Sample Number	Ingredients Listed on Food Labels on the Packages
1	Salted kimchi cabbage, radish, white rice paste, red pepper powder, salted anchovy sauce, spring onion, garlic, salted shrimp, ginger, sucrose, mushroom
2	Salted kimchi cabbage, radish, glutinous rice paste, garlic, red pepper powder, kelp extract, salted anchovy sauce, vegetables, salted shrimp sauce, spring onion, fermented vegetable lactic acid bacteria broth, onion, lactic acid bacteria broth, gelatinization rice powder, ginger
3	Salted kimchi cabbage, red pepper powder, garlic, radish, spring onion, onion, ginger, salt, sucrose, salted anchovy sauce, salted shrimp
4	Salted kimchi cabbage, radish, red pepper powder, fish sauce, kimchi seasoning paste, garlic, pear puree, spring onion, vegetables, salted shrimp sauce, spring onion, salted anchovy sauce, ginger, fermented lactic acid bacteria broth, crystalline fructose
5	Salted kimchi cabbage, radish, purified water, red pepper powder, rice powder, salted anchovy sauce, garlic, onion, spring onion, chives/leaf mustard, salted shrimp, water parsley, sucrose, pear, refined salt, chestnut, ginger, wheat extract, xanthan gum
6	Salted kimchi cabbage, radish, dried red pepper, garlic, onion, spring onion, salted anchovy sauce, salted shrimp, sucrose, ginger, red pepper powder, chives/leaf mustard, glutinous rice paste, refined salt, mushroom
7	Salted kimchi cabbage, radish, glutinous rice paste, red pepper powder, garlic, onion, salted anchovy sauce, salted shrimp, salted anchovy, spring onion, dried red pepper, chives/leaf mustard, kelp stock, ginger, sea staghorn
8	Salted kimchi cabbage, sliced radish, red pepper powder, garlic, spring onion, salted anchovy sauce, salted shrimp, onion, ginger, red pepper, sucrose, purified water, flour paste, refined salt, monosodium L-glutamate (flavor enhancer)
9	Salted cabbage kimchi, red pepper powder, anchovy sauce, radish, glutinous rice paste, salted shrimp, onion, garlic, onion, ginger, solar salt
10	Salted cabbage kimchi, radish, red pepper powder, garlic, salted anchovy sauce, salted sand lance sauce, spring onion, water parsley, onion, pear, paprika, salted shrimp, sucrose, glutinous rice paste, ginger, monosodium L-glutamate (flavor enhancer)
11	Kimchi cabbage, radish, red pepper powder, garlic, ginger, spring onion, leaf mustard/chives, onion, apple, salted shrimp, fermented anchovy sauce with meju (fermented soybean lump) powder, natural seasoning, glutinous rice paste, starch syrup, glucose, enzymatically modified stevia glucosyl stevia, kimchi lactic acid bacteria powder (*Leuconostoc mesenteroides*)
12	Kimchi cabbage, radish, red pepper powder, salted anchovy sauce, garlic, onion, salted shrimp, refined salt, chives, ginger, sucrose
13	Salted cabbage kimchi, radish, red pepper powder, garlic, salted shrimp, salted anchovy, ginger, onion, glutinous rice paste, sucrose

**Table 2:** Physicochemical properties of the 13 commercial kimchi samples.

Sample Number	pH	Total Acidity (%)	Salinity (%)	Free Sugars (mg/mL)	Free Amino Acids (mg/kg)
1	5.7 ± 0.0 ^e^	0.3 ± 0.0 ^c^	1.6 ± 0.0 ^f^	42.5 ± 0.8 ^f^	3562.7 ± 63.5 ^b^
2	6.1 ± 0.0 ^j^	0.2 ± 0.0 ^a^	1.5 ± 0.0 ^d^	41.7 ± 1.2 ^f^	4035.7 ± 148.1 ^c^
3	5.7 ± 0.0 ^f^	0.4 ± 0.0 ^e^	1.8 ± 0.0 ^i^	43.8 ± 3.5 ^f^	5122.4 ± 220.0 ^g^
4	4.3 ± 0.0 ^b^	0.8 ± 0.0 ^i^	1.3 ± 0.0 ^a^	25.8 ± 0.2 ^bc^	4345.5 ± 245.3 ^d^
5	4.6 ± 0.0 ^c^	0.5 ± 0.0 ^g^	1.5 ± 0.0 ^de^	27.3 ± 0.6 ^c^	3724.1 ± 66.5 ^b^
6	6.3 ± 0.0 ^k^	0.3 ± 0.0 ^b^	1.4 ± 0.0 ^c^	33.1 ± 1.1 ^d^	4205.7 ± 108.4 ^cd^
7	6.0 ± 0.0 ^i^	0.3 ± 0.0 ^d^	1.5 ± 0.0 ^e^	36.1 ± 1.0 ^e^	4363.6 ± 109.2 ^de^
8	5.1 ± 0.0 ^d^	0.4 ± 0.0 ^f^	1.4 ± 0.0 ^b^	32.1 ± 0.6 ^d^	4840.5 ± 161.7 ^f^
9	5.8 ± 0.0 ^g^	0.4 ± 0.0 ^e^	1.7 ± 0.0 ^h^	31.3 ± 0.6 ^d^	4637.3 ± 150.6 ^ef^
10	4.1 ± 0.0 ^a^	0.7 ± 0.0 ^h^	1.8 ± 0.0 ^h^	19.9 ± 0.7 ^a^	2855.6 ± 100.4 ^a^
11	5.9 ± 0.0 ^h^	0.3 ± 0.0 ^b^	1.5 ± 0.0 ^de^	37.4 ± 0.1 ^e^	4311.4 ± 19.9 ^cd^
12	4.6 ± 0.0 ^c^	0.7 ± 0.0 ^h^	2.0 ± 0.0 ^j^	23.8 ± 0.7 ^b^	4701.7 ± 121.9 ^f^
13	5.8 ± 0.0 ^g^	0.5 ± 0.0 ^g^	1.7 ± 0.0 ^g^	52.2 ± 0.5 ^g^	7682.1 ± 333.0 ^h^

^a–h^ Different small letters in a column indicate significant differences (*α =* 0.05) between mean values.

**Table 3:** Capsaicinoid content of 13 commercial kimchi samples.

Sample Number	Capsaicinoid Content ^1^ (mg/kg)	Spiciness Intensity Rating (15-Point Universal Scale in Spectrum^TM^)
1	9.0 ± 1.0 ^a^	1.7 ^a^
2	9.6 ± 1.1 ^a^	2.7 ^b^
3	9.8 ± 1.1 ^a^	2.2 ^ab^
4	10.3 ± 1.2 ^a^	1.9 ^a^
5	12.6 ± 1.1 ^b^	3.9 ^c^
6	12.7 ± 1.1 ^b^	2.4 ^ab^
7	14.4 ± 1.1 ^b^	2.0 ^ab^
8	16.9 ± 1.4 ^c^	3.5 ^bc^
9	17.7 ± 1.1 ^c^	3.4 ^bc^
10	20.3 ± 1.0 ^d^	5.2 ^d^
11	20.4 ± 1.5 ^d^	1.7 ^a^
12	26.7 ± 1.0 ^e^	6.7 ^e^
13	30.5 ± 1.0 ^f^	5.5 ^de^

^1^ Capsaicinoid content including capsaicin and dihydrocapsaicin. ^a–f^ Different small letters in a column indicate significant differences (*p* < 0.05) between mean values.

**Table 4:** Partial correlation coefficients showing the relationships among physicochemical properties, capsaicin contents, and rating of spiciness intensity.

	Pearson’s Correlation		Partial Correlation									
			pH ^3^		Titratable Acidity ^3^ (%)		Salinity ^3^ (%)		Free Sugars (mg/mL)		Free Amino Acids (mg/kg)	
	Cap ^1^	PIR ^2^	CAP	PIR	CAP	PIR	Cap	PIR	Cap	PIR	Cap	PIR
Cap	1.000	0.777	0	0.814 *** 0.001	0	0.762 ** 0.003	0	0.694 * 0.021	0	0.901 *** 0.009	0	0.778 *** 0.008
PIR	0.777	1.000	0.814 *** 0.001	0	0.762 ** 0.003	0	0.694 * 0.021	0	0.901 *** 0.009	0	0.778 *** 0.008	0

^1^ Capsaicinoid content (variable X); ^2^ spiciness intensity rating (variable Y); ^3^ condition variable Z. * *p* ≤ 0.05, ** *p* ≤ 0.01, *** *p* ≤ 0.001.

## Data Availability

The data presented in this study are available on request from the corresponding author.

## Supplementary Materials

The following are available online at https://www.mdpi.com/2304-8158/10/1/86/s1, Table S1: Free sugar contents (mg/mL) of 13 commercial kimchi samples, Table S2: Free amino acid contents (mg/kg) of 13 commercial kimchi samples.
