# Supplementary material for: Correlating Capsaicinoid Levels and Physicochemical Properties of Kimchi and Its Perceived Spiciness
Source: Foods. 2021 Jan 4;10(1):86. doi: 10.3390/foods10010086 (PMC7829842; doi:10.3390/foods10010086)
Supplement: Supplementary file 1 [file foods-10-00086-s001.pdf]

foods

MDPI

Supplementary Data

Table 1. Free sugar contents (mg/mL) of 13 commercial kimchi samples.

No	Free sugars (mg/mL)					
	**Glucose**	**Fructose**	**Sucrose**	**Maltose**	**Mannitol**	**Sorbitol**
1	19.0 ± 0.4^{g}	18.7 ± 0.8^{f}	4.7 ± 0.3^{d}	ND[1)]	ND	ND
2	17.5 ± 0.1^{f}	16.2 ± 0.7^{e}	ND	ND	ND	8.0 ± 0.4^{e}
3	18.8 ± 0.3^{g}	20.3 ± 3.7^{f}	4.0 ± 0.1^{c}	ND	0.6 ± 0.1^{b}	ND
4	8.0 ± 0.2^{a}	3.9 ± 0.0^{a}	ND	ND	10.3 ± 0.1^{g}	3.5 ± 0.1^{d}
5	12.8 ± 0.3^{c}	12.7 ± 0.1^{c}	ND	ND	1.7 ± 0.4^{c}	ND
6	13.9 ± 0.4^{d}	14.8 ± 0.0^{cde}	4.3 ± 0.7^{cd}	ND	ND	ND
7	15.5 ± 0.1^{e}	15.2 ± 0.6^{de}	2.6 ± 0.2^{b}	2.3 ± 0.3^{c}	ND	0.4 ± 0.0^{b}
8	14.3 ± 0.1^{d}	13.8 ± 0.4^{cd}	ND	ND	4.0 ± 0.1^{e}	ND
9	15.3 ± 0.5^{e}	14.5 ± 0.0^{cde}	ND	1.4 ± 0.2^{b}	ND	ND
10	8.3 ± 0.0^{a}	8.0 ± 0.2^{b}	ND	ND	3.5 ± 0.5^{d}	ND
11	21.2 ± 0.2^{h}	13.0 ± 0.0^{c}	ND	3.1 ± 0.2^{d}	ND	ND
12	9.9 ± 0.4^{b}	8.2 ± 0.2^{b}	ND	ND	5.6 ± 0.3^{f}	ND
13	18.6 ± 0.4^{g}	$20. \quad \pm 0.3^{f}$	ND	11.5 ± 0.5^{e}	ND	1.3 ± 0.2^{c}

[1)]Not detected. [a–h] There are significant differences ($p < 0.05$) in the mean values with different small letters in a column.

Table 2. Free amino acid contents (mg/kg) of 13 commercial kimchi samples.

Free amino acid (mg/kg)	1	2	3	4	5	6	7	8	9	10	11	12	13
Glutamic acid	264.7 ± 26.9^{a}	619.6 ± 4.9^{e}	1355.1 ± 79.8^{g}	522.8 ± 49.4^{d}	262.6 ± 26.8^{a}	283.7 ± 23.8^{a}	255.8 ± 36.7^{a}	1136.6 ± 15.4^{f}	241.2 ± 13.8^{a}	454.5 ± 11.7^{c}	377.3 ± 30.6^{b}	395.1 ± 19.7^{b}	2052.3 ± 44.1^{h}
Glutamine	754.8 ± 102.4^{bcd}	574.2 ± 89.5^{ab}	647.8 ± 62.2^{abc}	817.8 ± 84.9^{cd}	829.7 ± 110.1^{cd}	866.5 ± 129.5^{d}	881.4 ± 93.4^{d}	903.3 ± 148.0^{d}	583.1 ± 102.0^{ab}	523.9 ± 87.2^{a}	956.4 ± 128.4^{d}	820.8 ± 122.9^{cd}	972.4 ± 178.7^{d}
Phosphoserine	ND[x)]	ND	ND	ND	ND	ND	ND	ND	ND	ND	ND	ND	ND
Taurine	56.8 ± 0.9^{g}	33.4 ± 0.1^{bc}	46.9 ± 2.5^{e}	44.7 ± 2.9^{e}	32.3 ± 0.6^{b}	68.5 ± 0.3^{h}	50.3 ± 2.5^{f}	35.2 ± 0.3^{bcd}	67.4 ± 0.8^{h}	18.4 ± 0.3^{a}	36.4 ± 0.6^{cd}	37.2 ± 0.3^{d}	103.1 ± 3.7^{i}
Phosphoethanolamine	ND	ND	ND[1)]	ND	ND	ND	ND	ND	ND	ND	ND	ND	ND
Urea	ND	ND	ND	ND	ND	ND	ND	ND	ND	ND	ND	ND	ND
Aspartic acid	193.5 ± 4.2^{de}	157.2 ± 1.4^{b}	214.5 ± 12.8^{gh}	201.6 ± 12.6^{ef}	168.7 ± 3.9^{c}	206.3 ± 2.0^{fg}	220.4 ± 8.5^{h}	184.2 ± 1.1^{d}	197.4 ± 2.2^{ef}	97.4 ± 1.0^{a}	152.9 ± 2.0^{b}	238.8 ± 0.4^{i}	369.9 ± 3.0^{j}
Threonine	107.2 ± 1.6^{cd}	88.8 ± 1.7^{a}	109.2 ± 5.9^{cd}	143.6 ± 8.1^{f}	109.7 ± 1.9^{cd}	110.8 ± 0.6^{d}	127.2 ± 6.1^{e}	126.3 ± 0.8^{e}	103.4 ± 2.2^{bc}	83.0 ± 0.7^{a}	99.0 ± 1.2^{b}	131.0 ± 0.7^{e}	192.5 ± 3.4^{g}
Serine	141.6 ± 2.2^{ef}	113.6 ± 2.2^{b}	122.7 ± 6.4^{c}	147.5 ± 8.3^{fg}	133.3 ± 1.8^{d}	149.9 ± 0.4^{g}	182.5 ± 7.6^{h}	138.8 ± 1.0^{de}	111.4 ± 2.0^{b}	100.2 ± 0.6^{a}	141.0 ± 0.7^{ef}	151.6 ± 1.1^{g}	212.7 ± 5.7^{i}
Asparagine	247.7 ± 3.7^{de}	240.7 ± 3.5^{de}	241.9 ± 12.5^{de}	244.1 ± 13.0^{de}	188.7 ± 2.7^{a}	263.8 ± 0.6^{f}	203.1 ± 7.3^{b}	240.8 ± 2.7^{de}	235.9 ± 5.4^{d}	184.9 ± 1.6^{a}	220.4 ± 0.7^{c}	248.8 ± 3.8^{e}	346.6 ± 10.3^{g}
Sarcosine	ND	6.2 ± 5.4^{b}	ND	ND	ND	9.4 ± 0.3^{c}	ND	ND	ND	ND	ND	ND	ND
α-Aminoadipic acid	4.9 ± 0.8^{c}	7.2 ± 0.5^{d}	7.6 ± 0.8^{d}	9.9 ± 0.6^{f}	3.3 ± 0.2^{b}	10.1 ± 0.1^{f}	9.5 ± 0.8^{f}	8.3 ± 0.0^{e}	13.6 ± 1.2^{g}	ND	9.3 ± 0.6e^{f}	7.0 ± 0.1^{d}	12.8 ± 0.2^{g}
Glycine	73.3 ± 1.2^{b}	94.9 ± 2.6^{f}	82.1 ± 3.9^{c}	83.7 ± 4.5^{cd}	87.9 ± 0.8^{de}	111.4 ± 0.6^{h}	107.0 ± 4.6^{gh}	79.0 ± 0.9^{c}	147.3 ± 2.6^{j}	53.4 ± 0.7^{a}	89.8 ± 0.6^{e}	103.2 ± 0.8^{g}	130.4 ± 4.2^{i}
Alanine	334.8 ± 4.4^{d}	265.8 ± 6.7^{b}	288.2 ± 13.7^{c}	365.3 ± 20.1^{e}	327.1 ± 3.2^{d}	377.1 ± 1.7^{e}	417.3 ± 18.7^{f}	342.2 ± 3.5^{d}	502.1 ± 8.7^{i}	246.8 ± 2.5^{a}	443.3 ± 4.4^{g}	482.9 ± 3.9^{h}	544.8 ± 17.3^{j}
Citrulline	14.7 ± 0.1^{a}	25.7 ± 1.0^{bc}	56.7 ± 2.3^{f}	17.5 ± 1.8^{a}	32.5 ± 1.4^{d}	27.3 ± 0.6^{c}	31.3 ± 1.5^{d}	40.2 ± 1.6^{e}	27.1 ± 0.7^{bc}	24.1 ± 1.4^{b}	25.4 ± 0.5^{bc}	57.6 ± 1.1^{f}	77.7 ± 3.5^{g}
α-Amino-n-butyric acid	ND	40.2 ± 2.1^{b}	127.4 ± 8.6^{f}	81.6 ± 8.1^{e}	58.1 ± 3.8^{c}	41.6 ± 3.4^{b}	66.1 ± 4.2^{d}	65.4 ± 5.0^{d}	201.1 ± 3.1^{g}	ND	39.2 ± 0.8^{b}	132.1 ± 1.6^{f}	133.2 ± 3.7^{f}

Valine	142.4 ± 2.6^{cd}	148.1 ± 3.9^{d}	170.4 ± 10.0^{ef}	176.1 ± 11.1^{f}	163.3 ± 2.9^{e}	172.8 ± 1.5^{ef}	189.7 ± 8.2^{g}	118.0 ± 2.0^{b}	240.0 ± 5.2^{i}	105.5 ± 3.4^{a}	136.3 ± 1.0^{c}	209.8 ± 2.6^{h}	240.0 ± 8.2^{i}
Cysteine	ND	ND	ND	ND	ND	ND	ND	22.9 ± 39.7^{a}	ND	ND	51.2 ± 44.4^{b}	ND	ND
Methionine	45.1 ± 1.1^{cd}	43.1 ± 1.1^{cd}	51.2 ± 4.2^{e}	50.2 ± 4.1^{e}	41.5 ± 2.1^{c}	45.9 ± 1.0^{d}	52.8 ± 2.7^{ef}	35.3 ± 2.7^{b}	72.0 ± 1.5^{g}	25.3 ± 2.1^{a}	41.2 ± 0.5^{c}	56.7 ± 1.0^{f}	78.0 ± 2.4^{h}
Cystathionine	17.5 ± 1.2^{bcd}	23.2 ± 0.8^{e}	20.7 ± 3.5^{de}	19.3 ± 3.4^{cde}	13.1 ± 3.2^{a}	27.4 ± 0.3^{f}	14.9 ± 1.4^{ab}	19.4 ± 3.2^{cde}	18.9 ± 0.5^{bcd}	15.6 ± 2.9^{abc}	18.3 ± 0.6^{bcd}	18.1 ± 0.8^{bcd}	20.9 ± 0.8^{de}
Isoleucine	98.5 ± 1.7^{b}	96.3 ± 2.1^{b}	110.7 ± 8.0^{c}	107.1 ± 8.2^{c}	94.4 ± 3.7^{b}	111.7 ± 0.9^{c}	128.0 ± 6.3^{d}	91.4 ± 4.5^{b}	148.4 ± 2.9^{e}	61.3 ± 3.2^{a}	112.8 ± 1.4^{c}	146.3 ± 1.2^{e}	163.7 ± 5.7^{f}
Leucine	121.3 ± 2.1^{de}	109.8 ± 2.3^{bc}	131.5 ± 8.7^{f}	134.5 ± 9.9^{f}	117.1 ± 3.7^{cd}	127.0 ± 0.6^{ef}	157.1 ± 7.5^{g}	102.7 ± 4.4^{b}	165.7 ± 2.9^{g}	85.2 ± 3.5^{a}	131.1 ± 1.4^{f}	178.7 ± 1.5^{h}	209.2 ± 7.3^{i}
Tyrosine	62.5 ± 2.5^{bc}	57.8 ± 1.2^{bc}	68.1 ± 10.6^{cd}	77.8 ± 11.0^{de}	56.8 ± 10.2^{bc}	67.5 ± 0.8^{cd}	76.9 ± 5.5^{de}	53.9 ± 9.2^{b}	84.3 ± 1.4^{e}	40.8 ± 6.4^{a}	61.3 ± 1.1^{bc}	67.8 ± 0.8^{cd}	118.4 ± 5.2^{f}
Phenylalanine	75.2 ± 2.0^{bc}	71.1 ± 2.1^{bc}	87.3 ± 7.7^{de}	85.0 ± 8.6^{de}	79.1 ± 4.5^{cd}	90.0 ± 0.6^{e}	102.4 ± 5.3^{f}	69.0 ± 5.7^{b}	122.1 ± 3.1^{g}	50.9 ± 5.3^{a}	86.1 ± 1.3^{de}	101.2 ± 1.3^{f}	128.4 ± 5.9^{g}
β-Alanine	19.9 ± 2.1^{bc}	19.6 ± 1.1^{bc}	27.3 ± 5.5^{de}	23.1 ± 5.1^{cd}	16.1 ± 4.4^{b}	23.0 ± 1.1^{cd}	19.0 ± 0.7^{bc}	19.8 ± 0.9^{bc}	24.9 ± 0.9^{de}	10.2 ± 0.1^{a}	16.6 ± 0.0^{b}	25.4 ± 0.5^{de}	28.6 ± 1.7^{e}
β-Amino-n-butyric acid	ND	ND	ND	ND	6.7 ± 11.6^{b}	ND	ND	ND	ND	ND	ND	ND	ND
γ-Amino-n-butyric acid	155.8 ± 1.5^{a}	441.5 ± 10.0^{g}	394.9 ± 29.5^{f}	344.2 ± 32.8^{e}	264.8 ± 3.8^{c}	264.3 ± 2.9^{c}	269.4 ± 11.6^{c}	317.5 ± 0.5^{d}	319.5 ± 7.3^{d}	246.9 ± 2.9^{c}	211.0 ± 1.4^{b}	221.0 ± 2.2^{b}	360.1 ± 14.3^{e}
Ethanol amine	19.7 ± 1.3^{cd}	16.8 ± 0.3^{bc}	19.5 ± 6.2^{cd}	19.5 ± 5.8^{cd}	14.0 ± 0.4^{b}	19.5 ± 0.2^{cd}	18.8 ± 1.5^{cd}	17.6 ± 0.3^{bcd}	21.9 ± 0.3^{cd}	7.6 ± 0.1^{a}	17.6 ± 0.3^{bcd}	16.0 ± 0.6^{bc}	25.4 ± 0.2^{de}
Ammonia	73.7 ± 6.8^{b}	78.3 ± 3.4^{bc}	85.1 ± 12.4^{bcd}	113.3 ± 12.2^{fgh}	90.8 ± 8.8^{cde}	103.6 ± 8.2^{ef}	75.3 ± 11.3^{bc}	95.9 ± 9.3^{de}	98.2 ± 5.1^{def}	58.5 ± 6.7^{a}	104.4 ± 10.4^{efg}	120.0 ± 8.5^{gh}	122.3 ± 6.8^{h}
Hydroxylysine	ND	ND	ND	ND	ND	ND	ND	ND	ND	ND	ND	ND	ND
Ornithine	28.4 ± 0.6^{b}	25.4 ± 0.7^{b}	37.0 ± 1.6^{c}	202.5 ± 12.3^{f}	84.3 ± 0.9^{d}	28.4 ± 0.1^{b}	29.3 ± 1.3^{b}	26.6 ± 0.3^{b}	82.2 ± 1.6^{d}	16.2 ± 0.1^{a}	32.7 ± 6.3^{bc}	192.9 ± 1.6^{e}	84.1 ± 4.5^{d}
Lysine	112.4 ± 1.9^{b}	126.4 ± 3.2^{c}	146.8 ± 6.4^{ef}	149.0 ± 9.2^{f}	125.9 ± 1.4^{c}	133.8 ± 0.8^{cd}	164.1 ± 7.6^{g}	115.6 ± 1.2^{b}	277.3 ± 4.9^{h}	75.3 ± 0.7^{a}	140.6 ± 7.0^{de}	163.9 ± 1.4^{g}	273.5 ± 2.7^{h}
1-Methylhistidine	ND	ND	ND	ND	ND	ND	ND	ND	ND	ND	ND	ND	ND

Histidine	35.5 ± 0.8[c]	30.8 ± 0.7[b]	41.4 ± 1.9[e]	46.0 ± 2.7[g]	38.1 ± 0.3[d]	42.6 ± 0.2[ef]	52.7 ± 2.5[h]	34.9 ± 0.4[c]	58.4 ± 1.0[i]	26.4 ± 0.2[a]	42.7 ± 1.4[ef]	44.4 ± 0.5[fg]	52.3 ± 0.2[h]
3-Methylhistidine	ND	ND	ND	ND	ND	ND	ND	ND	ND	ND	ND	ND	ND
Anserine	ND	ND	ND	ND	ND	ND	ND	ND	ND	ND	ND	ND	ND
Carnosine	ND	ND	ND	ND	ND	ND	ND	ND	ND	ND	ND	ND	ND
Arginine	269.2 ± 4.41[g]	355.1 ± 7.1[j]	253.2 ± 12.9[e]	3.7 ± 0.29	139.3 ± 1.3[c]	294.2 ± 1.5[h]	279.7 ± 12.6[g]	277.8 ± 3.0[g]	261.8 ± 4.5[ef]	160.3 ± 1.4[d]	308.8 ± 2.8[i]	51.0 ± 0.6[b]	442.0 ± 14.2[k]
Hydroxyl proline	ND	ND	ND	ND	ND	ND	ND	ND	ND	ND	ND	ND	ND
Proline	90.4 ± 2.6[b]	124.9 ± 4.9[d]	176.9 ± 8.9[f]	113.1 ± 8.0[c]	143.6 ± 2.2[e]	126.4 ± 1.6[d]	180.3 ± 8.7[fg]	120.2 ± 0.8[cd]	209.3 ± 3.8[h]	81.9 ± 0.2[a]	206.9 ± 0.3[h]	280.9 ± 2.2[i]	185.6 ± 5.1[g]

[1)]Not detected. [a–k] There are significant differences ($p < 0.05$) in the mean values with different small letters in a column.
